# Adverse childhood experiences, psychological distress, and the role of locus of control

**DOI:** 10.3389/fepid.2026.1915843

**Published:** 2026-09-03

**Authors:** I. Behrens, B. Schmalbach, E. Brähler, V. Clemens, K. Petrowski, J. M. Fegert

**Affiliations:** 1 Medical Psychology & Medical Sociology, University Medical Center of the Johannes Gutenberg-University Mainz, Mainz, Germany; 2 Department of Psychosomatic Medicine and Psychotherapy, Medical Center of the Johannes Gutenberg University Mainz, Mainz, Germany; 3 Clinic and Polyclinic for Psychosomatic Medicine and Psychotherapy, Rostock University Medical Center, Rostock, Germany; 4 Department of Child and Adolescent Psychiatry and Psychotherapy, Ulm University Medical Center, Ulm, Germany

**Keywords:** adverse childhood experiences, control beliefs, locus of control, mental health, psychological distress

## Abstract

Adverse childhood experiences (ACEs), including abuse, neglect, and household dysfunction, are prevalent and represent a major risk factor for psychological distress in adulthood. Although control-related constructs such as self-efficacy, mastery, and perceived control have been widely examined in this context, the role of locus of control (LoC) remains less well understood. The present study investigated the associations between ACEs, LoC, and psychological distress, and examined whether LoC mediates or moderates the relationship between ACE exposure and psychological distress. Data were drawn from a representative survey of the German general population [*N* = 2,463; age range 16–96 years; female 1,237 female (50.2%)]. ACEs were assessed using the Adverse Childhood Experiences Questionnaire, psychological distress was measured with the Patient Health Questionnaire–4, and LoC was assessed with the Internal–External Locus of Control Short Scale–4. Structural equation modeling with robust diagonally weighted least squares estimation was applied. Higher ACE exposure significantly predicted greater psychological distress and a more external LoC orientation. Mediation analyses indicated that LoC accounted for approximately half of the association between ACE exposure and psychological distress. In contrast, moderation analyses did not reveal a significant interaction effect between ACE exposure and LoC in predicting psychological distress. These findings highlight control beliefs as an important psychological factor, linking early adversity to adult psychological distress and suggest that interventions targeting perceived control may help mitigate long-term mental health consequences of childhood adversity.

## 1 Introduction

Adverse Childhood Experiences (ACEs)—including abuse, neglect, and household dysfunction—are common and strongly linked to poor psychological health. In Germany, 43.7% of adults report at least one ACE (1), which elevates the risk of depression and anxiety (2), with affected individuals facing a seven- to eightfold higher risk for these disorders (1). Psychological distress, often manifesting as depressive and anxiety symptoms such as hopelessness, worry, and sleep difficulties (3), may stem from perceived lack of control (4).

ACEs encompass chronic or severe childhood events that harm or distress the child and disrupt development (5). They include active maltreatment (e.g., abuse), neglect, and household dysfunction (e.g., parental substance abuse, mental illness, domestic violence, separation/divorce, or incarceration). Unlike narrower definitions of childhood trauma, ACEs cover this broader range and vary in severity, harm, distress, and cumulativeness.

Locus of control (LoC), introduced by Rotter (6), describes whether individuals attribute outcomes to internal (self-driven) or external (chance/others) factors. Internal LoC fosters proactive coping, while external LoC is associated with passivity and greater emotional distress (7, 8). Although similar to mastery, LoC has received less attention in ACE research than self-efficacy or mastery.

Extensive evidence, including meta-analyses and longitudinal studies, shows a dose-response relationship: greater ACE exposure predicts higher depression and anxiety (9, 10). ACEs are also linked to lower self-efficacy and mastery (11, 12), indicating reduced agency. Limited LoC-specific studies suggest ACEs (or negative life events) correlate with more external LoC (13), possibly as an adaptive response to uncontrollable early environments. Per Kay et al. (14) compensatory control model, external sources of control may protect perceived order under threat, specifically in childhood but become maladaptive in adulthood, increasing vulnerability. In particular, the outsourcing of personal control over a given situation to an external entity fits well with the LoC framework.

Learned helplessness theory (15, 16) suggests repeated uncontrollable events like ACEs foster helplessness and distress, which internal LoC may buffer and external LoC may amplify. While self-efficacy and mastery have been examined as mediators/moderators (17), LoC is understudied. One study found general perceived control moderated the ACE–distress link, but used non-LoC-specific measures and a non-representative student sample (18).

Overall, evidence indicates LoC may moderate the ACE–psychological distress relationship, with internal LoC potentially protective and external LoC increasing vulnerability. This study investigates this hypothesis with more targeted, representative methods.

Based on the state of research, the hypotheses for this study can be derived from the established links between ACEs, psychological distress, and LoC. Previous studies have robustly demonstrated that ACEs are associated with increased psychological distress (2, 9, 10, 19–25). This relationship is characterized by consistent effect sizes across diverse methodologies and populations (9), suggesting that individuals with ACEs show significantly higher levels of distress compared to those without such experiences. Accordingly, this study aims to replicate the established association between ACE exposure and psychological distress. Therefore, the first hypothesis (H1) posits:

H1: Individuals with higher ACE exposure report higher levels of psychological distress.

As described above, the impact of ACEs on control beliefs such as self-efficacy and mastery has been more thoroughly researched than its influence on LoC. Studies have shown that ACEs are associated with diminished self-efficacy and mastery (11, 26, 27). Nontheless, LoC provides distinct added value. While self-efficacy focuses on perceived capability to perform specific behaviors and mastery emphasizes skill acquisition, LoC specifically addresses generalized beliefs about whether outcomes are determined by one's own actions (internal) or by external forces (external) (6). Empirically, LoC offers unique explanatory power for persistence, coping, and behavioral outcomes beyond self-efficacy and mastery.

Existing evidence regarding LoC suggests that ACEs may contribute to shifts in LoC toward a more external and less internal orientation. For example, Hovenkamp-Hermelink et al. (13) found that negative life events predicted a more external LoC, while positive life events were linked to a more internal LoC. These findings support the following hypothesis:

H2a: Individuals with higher ACE exposure exhibit a more external LoC.

Certain constructs like perceived social support and coping strategies have been shown to mediate or moderate the relationship between ACEs and psychological distress (17). Similarly, LoC has emerged as another critical mechanism, functioning both as a predictor and mediator in the ACEs-psychological distress relationship (7, 17).

H2b: Individuals with a more external LoC exhibit higher psychological distress.

Individuals with a more external LoC attribute outcomes to luck, fate, or powerful others. As a result they experience lower personal agency, maladaptive coping, and learned helplessness, which in turn are associated with higher psychological distress.

H3: LoC mediates the relationship between ACEs and psychological distress.

While the mediating role of LoC has already been established, the moderating effect of LoC has yet to be thoroughly investigated. The findings of Davis and Burrow (18) provide preliminary evidence, suggesting that external LoC may exacerbate symptoms following ACEs, while internal LoC may buffer these effects. Nevertheless, this study was limited by its non-representative sample and reliance on the Sense of Control Scale (28), which, while conceptually consistent with LoC, does not explicitly measure fatalistic external LoC constructs such as fate or chance. These limitations emphasize the need for further research using representative populations and LoC-specific measures to confirm these findings. Thus, the following hypotheses were formulated:

H4: LoC moderates the relationship between ACEs and psychological distress, with more external LoC amplifying the association and more internal LoC mitigating it.

These hypotheses aim to replicate previous findings on the effects of ACEs on psychological distress and control beliefs, as well as extend existing research by investigating the moderating role of LoC between ACEs and psychological distress, using a representative sample of the German population.

## 2 Methods

### 2.1 Sample and procedure

The present study employed a representative sample of the German general population, recruited with the support of the demographic research institute USUMA (Berlin, Germany) in 2022. The total number of participants was *N* = 2,522. Participants who provided complete information on the main variables, ACEs, LoC, as well as psychological distress, were included in the analysis of this study. After the exclusion of incomplete data sets, the sample comprised *N* = 2,463 participants. Participants' ages ranged from 16 to 96 years. A further selection of socio-demographic characteristics of the sample is shown in Table 1. Sampling followed a multistage random-route procedure based on electoral districts, complemented by the Kish selection grid to ensure unbiased participant selection. This methodology yielded a demographically representative sample with respect to sex, age, and educational attainment. Eligibility required sufficient proficiency in the German language. Sampling points were allocated disproportionately, comprising 210 in the former West German states and 96 in the new federal states, consistent with the ADM master sample framework. Within each randomly chosen household, the target respondent was identified using the so-called “Kish selection grid” which randomly selects individuals from the household roster. Nonresponding households were recontacted at least twice at varying times before being excluded from the sample.

**Table 1:** Sociodemographic information on the sample.

Variable	*N* = 2,463	
	*M*	*SD*
Age	49.29	17.66
	*n*	%
Gender		
Female	1,237	50.22
Male	1,222	49.62
Non-binary	4	0.16
Family status		
Single	716	29.09
Married	1,179	47.90
Separated	355	14.43
Widowed	211	8.57
Not stated	2	0.08
Children (below 16 years old)		
Yes	488	19.81
No	1,975	80.19
Education (highest degree)		
No degree	58	2.36
Lower secondary school	603	24.52
Middle secondary school	828	33.67
Polytechnical School	208	8.46
Technical training	108	4.39
High school	324	13.18
University degree	284	11.55
Other	6	0.24
Still in school	40	1.63
Not stated	4	0.16
Household income		
Low Income	739	35.75
Middle/ Moderate Income	602	29.12
High Income	692	33.48
Not stated	34	1.65

Trained interviewers conducted home visits to obtain informed consent and gather sociodemographic data. Participants subsequently completed several standardized self-report questionnaires. Prior to data collection, interviewers explained the study aims, procedures, and data protection measures, and each participant received a written privacy statement. No invasive, medical, or psychologically harmful procedures were part of the research, and therefore, according to German ethical regulations, verbal informed consent was deemed sufficient. All procedures complied with the ethical principles outlined in the Declaration of Helsinki (revised 2008) and the International Committee of Medical Journal Editors (ICMJE) Recommendations for the Protection of Research Participants. The study procedure was approved by the ethics committee of the medical faculty of the local university (#594/21-ek).

### 2.2 Instruments

#### 2.2.1 Adverse childhood experiences questionnaire (ACE-Q)

ACEs were assessed using the German version of the Adverse Childhood Experiences Questionnaire ACE-Q (29). The 10-item measure captures experiences of abuse, neglect, and household dysfunction (e.g., emotional, physical, and sexual abuse; parental separation; substance abuse; mental illness). Items are answered dichotomously (“yes”/“no”) and summed to yield a total score (0–10). ACEs were primarily operationalized as a continuous sum score (range 0–10), with higher scores indicating greater exposure. For descriptive purposes and sensitivity analyses, ACE exposure was additionally dichotomized (0 vs. ≥ 1 ACE), consistent with prior research. Internal consistency was good (*α* = 0.79). Construct validity with the Childhood Trauma Questionnaire has been reported as high r = .84 (29).

#### 2.2.2 Patient health questionnaire–4 (PHQ-4)

Psychological distress was measured with the German version of the Patient Health Questionnaire–4 PHQ-4 (30–32), a brief screening tool for depression and anxiety. Participants rated four items (e.g., “Feeling nervous, anxious or on edge”) on a 4-point scale from 0 (“not at all”) to 3 (“nearly every day”). Total scores range from 0 to 12, with higher scores indicating greater distress. The scale demonstrated excellent reliability in the present sample (α = .88).

#### 2.2.3 Internal–external locus of control short scale–4 (IE-4)

LoC was assessed using the Internal–External Locus of Control Short Scale–4 IE-4 (33). The scale comprises four items on a 5-point Likert scale (1 = “strongly disagree” to 5 = “strongly agree”). Although the scale was originally conceptualized with internal and external LoC as two separate dimensions, recent psychometric work suggests that LoC may be adequately represented as a single bipolar construct (34). Accordingly, LoC was modeled as a unidimensional latent factor, with higher scores reflecting a more internal and lower scores reflecting a more external control orientation. Internal consistency was good in the present sample with ω = .806.

#### 2.2.4 Transparency and openness

All data, analysis code, and research materials are available upon request from the first author. Neither the study design nor the analysis plan were pre-registered. We report how we determined our sample size, all data exclusions (if any), all manipulations, and all measures in the study. The sample size was determined with the goal of representativeness (not statistical power). Specifically, we aimed for a sample size of at least 2,401 based on a population of 83 million, a margin of error of 2%, and confidence level of 95%.

### 2.3 Statistical analyses

All analyses were conducted using R and the packages lavaan and modsem (35, 36). We utilized structural equation modeling (SEM) to analyze the latent constructs under study—rather than the mere observed scores. All three were investigated as latent variables. We employed robust diagonally weighted least squares estimation (WLSMV in lavaan) because of the ordinal nature of the items. We evaluated model fit using the typical fitindices and the common cutoffs CFI/TLI >.95, RMSEA/SRMR >.08 (37). To test for measurement invariance between individuals with versus without ACEs reported, we compared *χ*^2^, CFI and calculated the RMSEAD according to the recommendations by Savalei et al. (38): CFI should be smaller than.01, wheras RMSEAD should be smaller than.08. The mediation analysis was then also conducted in SEM to estimate the direct and indirect effects.

For the moderation analysis, we utilized the *modsem* package, which builds on lavaan but allows for interactions between latent variables. Specifically, we utilized the product indicator approach *dblcent*. Because of convergence issues, we were not able to estimate the model using WLSMV but instead used MLR.

## 3 Results

### 3.1 Descriptive analysis

An overview of the sample's demographic characteristics is provided in Table 1. Before the inferential statistics were carried out, an overview of the distributions of the main variables in the sample was created. Table 2 presents descriptive statistics for psychological distress and LoC, including means and standard deviations—along with the potential range for each score. In addition, the table provides descriptive information on ACE exposure across increasing numbers of ACEs.

**Table 2:** Information on the distributions of psychological distress and locus of control (LoC) in the sampl*e.*

Questionnaire	*N* = 2,463	
	*M*	*SD*
Total PHQ-Score (0–12)	1.41	2.12
LoC (4–20)	12.90	1.84
Adverse Childhood Experiences (0–12)	0.87	1.60

	*N*	*%*
Number of ACEs		
Zero	1,643	66.71
One or more	820	33.29

### 3.2 Predictive value of ACE

For hypothesis 1, ACE was found to be a strong predictor of PHQ, β = .514 [.461,.567], *p* < .001. In addition, we compared latent PHQ scores between groups of individuals that reported zero ACEs versus one or more ACEs. To this end, we first established measurement invariance by constraining thresholds, loadings and item intercepts to be equal between groups—which led to a negligible decrease in model fit, *Δχ*^2^ = 11.36, *Δdf* = 10, *p* = .330, *ΔCFI* = .000, *RMSEA*_D_ = .007. The difference in PHQ latent means was then significant between groups, *d* = 0.968 [.881, 1.055], *p* < .001.

We conducted the equivalent analyses to test hypothesis 2a. Specifically, we found that ACE was a strong negative predictor of LoC, β = -.457 [.404,.510], *p* < .001. Again, we compared latent LoC scores between groups of individuals that reported zero ACEs versus one or more ACEs. Measurement invariance was confirmed by acceptable fit decay upon introducing equality constraints, *Δχ*^2^ = 80.79, *Δdf* = 13, *p* < .001, *ΔCFI* = .010, *RMSEA*_D_ = .046. The difference in LoC latent means was then significant between groups, *d* = 0.548 [.464,.632], *p* < .001.

Similarly, we were able to confirm hypothesis 2b, where we found that LoC was strongly associated with PHQ, *β* = -.669 [.626,.712], *p* < .001.

### 3.3 Mediation analysis

As demonstrated above, the primary relationship between ACE and PHQ is significant, with a large effect size. Subsequently, we tested to what extent this relationship can be explained in mediation by LoC. The fit of the latent variable mediation model was good, *χ*2(130) = 521.61, *p* < .001, *CFI* = .986, *TLI* = .984, *RMSEA* = .035, *SRMR* = .063. The individual paths are displayed in Table 3. In summary, there was a significant mediation effect via LoC which made up about half of the total effect of ACE on PHQ.

**Table 3:** Analysis of the mediating effect of locus of control (LoC) on the relationship between adverse childhood experiences (ACEs) and current psychological distress (PHQ*).*

Path	*b*	*SE*	*p*	*β* [95% *CI*]
ACE → LoC (a)	−.381	.027	<.001	−.459 [−.406, −.512]
LoC → PHQ (b)	−.621	.029	<.001	−.549 [−.492., −.606]
ACE → PHQ (c, direct effect)	.245	.029	<.001	.261 [.204,.318]
Indirect effect (a X b)	.236	.018	<.001	.252 [.217,.287]
Total effect (c + a X b)	.477	.027	<.001	.513 [.460,.566]

We obtained similar results when treating ACE as a binary predictor (none versus at least one ACE): Initially, ACE group predicted PHQ, *β* = .331 [.243,.419], *p* < .001, with a moderate effect size. We then tested for mediation by LoC. The fit of the latent variable mediation model was good, *χ*2(5) = 45.43, *p* < .001, *CFI* = .997, *TLI* = .997, *RMSEA* = .057, *SRMR* = .016. We display the individual paths in Table 4. In all, LoC mediated about half of the effect of ACE group on PHQ.

**Table 4:** Analysis of the mediating effect of locus of control (LoC) on the relationship between groups of adverse childhood experiences (ACE; 0 vs. 1+ ACEs) and current psychological distress (PHQ*).*

Path	*b*	*SE*	*p*	β [95% *CI*]
ACE → LoC (a)	−.702	.024	<.001	−.617 [−.570, −.664]
LoC → PHQ (b)	−.455	.039	<.001	−.262 [−.186, −.338]
ACE → PHQ (c, direct effect)	.335	.041	<.001	.169 [.089,.249]
Indirect effect (a X b)	.319	.029	<.001	.161 [.104,.218]
Total effect (c + a X b)	.655	.045	<.001	.331 [.243,.419]

When controlling the initial analysis (with count-based ACE) for the influence of age, gender, and income group, the results remain largely unchanged. Model fit was still good, *χ*2(178) = 726.81, *p* < .001, *CFI* = .975, *TLI* = .979, *RMSEA* = .039, *SRMR* = .063, and the indirect effect remained of moderate size at *β* = .230 [.193,.279], *p* < .001.

### 3.4 Moderation analysis

The latent moderation model of number of ACE and IE-4 predicting PHQ had unacceptable model fit with only the *RMSEA* indicating close fit, *χ*2(1,573) = 12,782.97, *p* < .001, *CFI* = .468, *TLI* = .441, *RMSEA* = .054, *SRMR* = .160. This makes interpreting parameter estimates questionable. The interaction term was significant, β = .036 [.003,.061], *p* = .032, but exhibited a negligible effect size. Accordingly the *R*^2^ change between the baseline model and the interaction model was.001.

When we redid the analyses using ACE groups (0 vs. 1+ ACEs), the results were quite similar. The model did not fit the data well, *χ*2(57) = 2,682.48, *p* < .001, *CFI* = .656, *TLI* = .529, *RMSEA* = .136, *SRMR* = .547. Similarly, the interaction term was significant, *β* = .061 [.034,.088], *p* < .001, but small and questionable in its interpretation. *R*^2^ change was small at.003.

For the sake of completeness, we also report the initial moderation analysis with covariates included (age, gender, income): Model fit was again unacceptable with the exception of *RMSEA*, *χ*2(1,744) = 12,312.29, *p* < .001, *CFI* = .476, *TLI* = .451, *RMSEA* = .055, *SRMR* = .155. The interaction term increased slightly when controlling for these three variables, β = .047 [.009,.085], *p* = .016. *R*^2^ change was again very low at.001.

## 4 Discussion

The present study examined associations between ACEs, locus of control (LoC), and psychological distress. Consistent with prior evidence, ACEs showed a robust association with psychological distress: higher exposure predicted significantly higher distress levels. This aligns with research demonstrating ACEs as strong predictors of depression and anxiety, with a consistent dose-response relationship between childhood adversity and adult psychological difficulties (1, 2, 9, 10). These results reinforce the long-term consequences of early-life stress on emotional well-being.

Moreover, higher ACE exposure was robustly associated with a more external LoC orientation. This mirrors findings on related constructs such as mastery and self-efficacy (11, 27) and is consistent with compensatory control theory (14), which suggests that uncontrollable environments lead individuals to attribute control externally in order to restore beliefs of controllability. While potentially adaptive in childhood, this orientation can become maladaptive in adulthood, contributing to poorer mental health outcomes.

Importantly, LoC mediated approximately half of the total effect of ACEs on psychological distress, underscoring control beliefs as a central psychological factor linking early adversity to later distress. This corroborates prior evidence on mastery and self-efficacy as key mediators in similar pathways (11, 12) and suggests diminished perceptions of control are crucially associated with ACEs' enduring impact.

Contrary to expectations, LoC did not reliably moderate the relationship between ACEs and psychological distress. We did find a significant *p* value when evaluating moderation effect. However, the associated effect size was negligibly small. In addition, model fit was unacceptable, putting into question the obtained parameter estimates. This diverges from Davis and Burrow (18), who found significant moderation effects of perceived control on ACEs' links to depression and anxiety. Methodological differences could explain this: they used the Sense of Control Scale (28), analyzed depression and anxiety separately, included multiple covariates (e.g., perceived stress, desire for control, resilience), and sampled US undergraduates. Particularly the focus on an overall sense of control as opposed to the locus of control stands out: Having a sense of control—be it internally or externally based—might help modulate the influence of ACEs on today's states. We hypothesize that the influence of ACEs on the specific locus of control is more state-like and stable, while overall sense of control seems more malleable via interventions. A future study should disentangle these two related processes.

The findings integrate theoretical frameworks on adversity and control. They complement learned helplessness theory (15, 16), which posits that repeated uncontrollable stressors foster generalized helplessness and reduced motivation. They also support expanding compensatory control theory (14) to ACEs, providing deeper insight into how early uncontrollable environments shape lifelong control beliefs and mental health trajectories.

Strengths include the large, representative sample with a wide age range. Limitations include the cross-sectional design, which precludes causal inferences (longitudinal studies are needed); brief screening instruments for ACEs and LoC, limiting precision; the traditional ACE framework's omission of stressors like parental loss, peer victimization (e.g., bullying), or other social adversities (39); restricted generalizability beyond the German context; and lack of other variables such as social support or resilience, which prior work suggests may be even stronger mediators (12). Including these in future research would be valuable.

What is more on the topic of generalizability, we found somewhat lower values for ACEs compared to previous studies: Witt and colleagues (1) found a prevalence rate of 43.7% and Schulz et al. (40) found 37.4%. It should be noted that both of these studies as well as our present investigation are based on data from representative surveys of the German general population (actually by the same survey company and the same research group led by Elmar Brähler). Since the methodology is identical, it stands to reason that the cause of the different prevalence rate are changes in the population, either in terms of fewer ACEs or—what seems more likely—a change in response behavior.

Practically, interventions strengthening internal control beliefs—via cognitive-behavioral or resilience-based approaches—may help mitigate distress in individuals with ACE histories. However, the lack of moderation indicates LoC changes alone may not buffer ACEs' effects sufficiently. Comprehensive, multi-component interventions addressing broader mechanisms (social support, emotional regulation, coping strategies) are likely necessary.

In summary, the study confirms ACEs' detrimental effects on psychological distress and control beliefs, identifying LoC as playing an important intervening role. These findings highlight the importance of addressing control beliefs in preventive and therapeutic interventions for early adversity exposure, while viewing LoC within a broader integrative framework of psychological and social determinants.

## Data Availability

The raw data supporting the conclusions of this article will be made available by the authors, without undue reservation.
